# Mortality of female and male Croatian Olympic athletes: a 1948-2016 cohort study

**DOI:** 10.3325/cmj.2025.66.309

**Published:** 2025-10

**Authors:** Vedran Radonić, Mario Šekerija, Marijan Erceg, Ivana Jurin, Irzal Hadžibegović, Miran Martinac, Tomislav Letilović

**Affiliations:** 1 Department of Cardiology, University Hospital Merkur, Zagreb, Croatia; 2 Croatian National Institute of Public Health, Zagreb, Croatia; 3 Croatian Institute of Public Health, Zagreb, Croatia; 4 Department for Cardiovascular Diseases, University Hospital Dubrava, Zagreb, Croatia; 5 Abdominal Surgery Department, University Hospital Merkur, Zagreb, Croatia; 6 University of Zagreb School of Medicine, Zagreb, Croatia

## Abstract

**Aim:**

To compare the overall mortality of Croatian male and female Olympic athletes who represented Yugoslavia or Croatia in the Olympic Games from 1948 to 2016 with the mortality of the general Croatian population standardized by age, sex, and time period.

**Methods:**

Overall mortality analysis included 652 male and 158 female Olympians. The cause-specific analysis included 642 male Olympians; due to the small sample size, this analysis was not possible for female Olympians. Information on causes of death was obtained from specialized registers of countries where deaths occurred, if available. Alternatively, data were obtained by interviews with families or acquaintances of the deceased, based on the World Health Organization verbal autopsy principles. Croatian general population mortality data were obtained from the Croatian Bureau of Statistics and World Health Organization’s databases. Overall and disease-specific standardized mortality ratios (SMR) were calculated.

**Results:**

At the endpoint of the study, 2 female and 142 male Olympians had died. Overall mortality was lower for women (SMR 0.23; *P* = 0.013) and men (SMR 0.56; *P* < 0.001) compared with the general population. Male Olympians had significantly reduced mortality from cardiovascular (SMR 0.51; *P* < 0.001), neoplastic (SMR 0.55; *P* < 0.001), respiratory (SMR 0.24; *P* = 0.003), and digestive (SMR 0.42; *P* = 0.015) causes of death.

**Conclusions:**

Croatian male and female Olympians experience reduced overall mortality compared with the Croatian general population. Croatian male Olympians have reduced cardiovascular, neoplastic, digestive, and respiratory mortalities compared with the Croatian general population.

Physical activity is widely acknowledged for its positive impact on health (1,2). Beyond its role in reducing overall mortality, substantial evidence supports its effectiveness in diminishing the prevalence of obesity, type 2 diabetes, arterial hypertension, atherosclerosis, coronary artery disease, certain malignancies, depression, and dementia (1,3-6).

On the other hand, the impact of elite sports on human health remains debatable (7-10). A pertinent question is the threshold of intensity and volume of physical activity beyond which it becomes excessive and potentially deleterious to health in the long term. While elite sports do not negate the health benefits of physical activity, high-level athletic practice is associated with an array of cardiovascular, neurological, psychiatric, and other disorders (11-15). Fundamental knowledge about the elite athletes’ survival compared with the general population has been obtained from local observational studies and respective meta-analyses. However, despite the existing data, elite athletes form a complex heterogeneous population with consequential analytic variations and limitations. Thus, studies about them still usually call for more research. Although general and cause-specific (mostly cardiovascular and neoplastic) survival benefits for elite athletes are demonstrated, there are occasional opposite findings across populations of different geographical regions and sport types (7-11,15-27). Furthermore, studies that include cause-specific mortality analyses are less common, while studies about female athletes, especially with long follow-up, are exceptionally rare (10,19-23,26). Notably, living conditions of the general population vary across countries, influencing differential rates of total and specific mortality. For instance, in 2014 Croatia displayed higher mortality rates relative to the European Union (EU) average across the three primary causes of death: cardiovascular diseases, neoplasms, and external causes, with all the age and sex groups taken into account together (28).

Olympians are regarded as pinnacle athletes, given that the Olympic Games (OG) represent the zenith of sports competition. United States (US), French, Polish, and Japanese Olympic athletes were demonstrated to have reduced mortality compared with their respective general populations, while no significant mortality advantage was found among German and Danish Olympic athletes (17,21,25,26,29-31). Only US and French authors provided information about female athletes’ general mortality, reporting their survival benefit, while US, French, and Danish studies provided data about cause-specific mortality (21,26,31). The literature on Olympic and other elite athletes shows significant geographic disparities. While the USA and Northern, Central, and Western Europe are relatively well represented, many other regions, including the countries of the former Yugoslavia and Southeastern Europe, lack high-powered studies (7-10,18-23,26,27). The region is represented by only one small-sample study on Croatian male Olympic medalists, who had reduced general and cardiovascular mortality rates (24). The aim of this study is to investigate overall and, whenever possible, disease-specific mortality among all Croatian Olympic athletes, incorporating sub-analyses based on age, sex, and calendar periods.

## PARTICIPANTS AND METHODS

### Participants and data collection

This study enrolled Croatian male and female athletes who competed in the summer or winter OG from 1948 to 2016 representing Yugoslavia (until 1988) or Croatia (1992 and onwards). The primary source of data regarding Croatian Olympians was the chronicle by the Croatian Olympic Committee (COC) entitled Croatian Olympians and Medalists (32). Published in September 2016, this source lists all Croatian Olympic athletes. Although today's Croatia was part of Yugoslavia through the majority of the follow-up period, not all the Yugoslav athletes are considered Croatian athletes. The COC recognized some of the Yugoslav Olympians as Croatian Olympians based on their internal criteria that rely on birthplace, ethnicity, and regional origin, and sports development within Croatian clubs or environment. Only athletes classified as Croatian by the COC were included in this study; former Yugoslav Olympic athletes not meeting this criterion were excluded. The COC chronicle served as the primary source for the Olympians' vital status, providing their dates of birth and, where applicable, death. The chronicle also explicitly noted any cases where an athlete's vital status was unknown.

The primary source for determining athletes’ causes of death were the official registers of the countries in which they died. For those who died in Croatia, data were obtained from the Croatian Institute of Public Health (CIPH) mortality database if available. Unfortunately, CIPH does not track causes of death data for people who died in today's Croatian territory when it was part of Yugoslavia. For athletes who died abroad, institutions from the respective countries were contacted, and data about causes of death were provided if available and if it was possible according to their internal regulations. Where data could not be obtained from official databases, national and regional sports associations and athletes' former sport clubs were engaged to assist the authors in establishing contact with the family members or acquaintances of the deceased athletes. If contact was successfully established, interviews were conducted using the World Health Organization (WHO) verbal autopsy (VA) principles. VA is a validated method used to retrospectively ascertain the causes of death for individuals lacking official postmortem documentation, with standardized guidelines provided by the WHO (33-36).

The general population mortality rates were calculated for each year as the ratio of documented deaths to the total population within the specified reference groups, standardized by sex, age, and period. Data on overall and cause-specific deaths documented in the Croatian general population during the period 1948-2016 were collected from the Croatian Bureau of Statistics, stratified by 10-year age intervals beginning at 15-24 years (37). Total Croatian population estimates from 1948 to 2016 for the same age groups were obtained from the official WHO website (38,39).

### Study design

This retrospective cohort study compared overall and cause-specific mortality of Croatian Olympic athletes and the general Croatian population standardized by age, sex, and period. Causes of death were categorized according to International Classification of Diseases (ICD)-10 codes, with cardiovascular diseases defined as I00-I99, malignant neoplasms as C00-C97, and external causes as V01-Y89. All remaining ICD-10 diagnoses were grouped under “other causes of death” (39). Since the analyses focused on broader cause-of-death groups rather than exact diagnoses, the potential for misclassification in cases where official documentation was unavailable was minimized. Analyses were conducted separately for male and female athletes. Athletes with undetermined vital status throughout the follow-up period were excluded from the study. Among the 653 male and 159 female athletes listed, only one male and one female athlete had an undetermined vital status at the end of the follow-up period. Thus, 652 male and 158 female athletes were included in this study. The low sample size for the female cohort precluded cause-of-death, age, and period-specific analyses, so the analysis was restricted to overall mortality determination.

**Figure 1:**
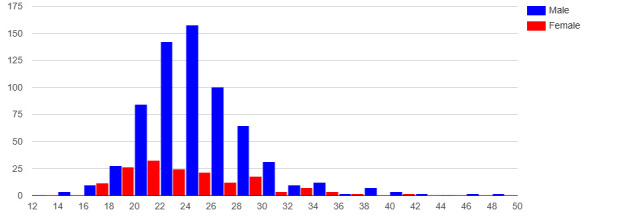
Age at first Olympic Games appearance for male and female Croatian Olympians. Age (years) is shown on the x-axis, and the number of athletes on the y-axis.

**Figure 2:**
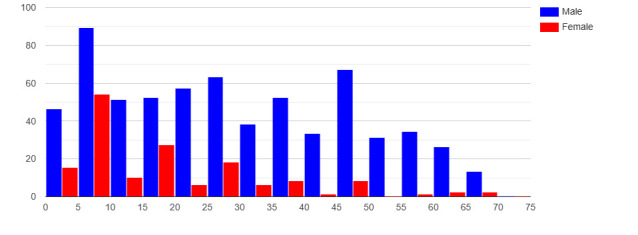
Follow-up duration for male and female Croatian Olympians. Follow-up time (years) is shown on the x-axis, and the number of athletes on the y-axis.

Ten deceased male athletes whose cause of death could not be ascertained through the defined methodology were excluded from the cause-specific mortality analysis. They were, however, included in the overall mortality analysis. Hence, 642 male athletes were used for the cause-specific mortality calculations. Given the acceptable proportion of missing data, we do not expect it to have introduced any material bias into the results. The follow-up for the athletes began on the first day of the year of their initial OG appearance and extended until the date of death or August 22, 2016, which served as the date of administrative censoring. The athletes’ age at their first OG appearance is shown in Figure 1, while the follow-up duration is shown in Figure 2.

**Figure 3:**
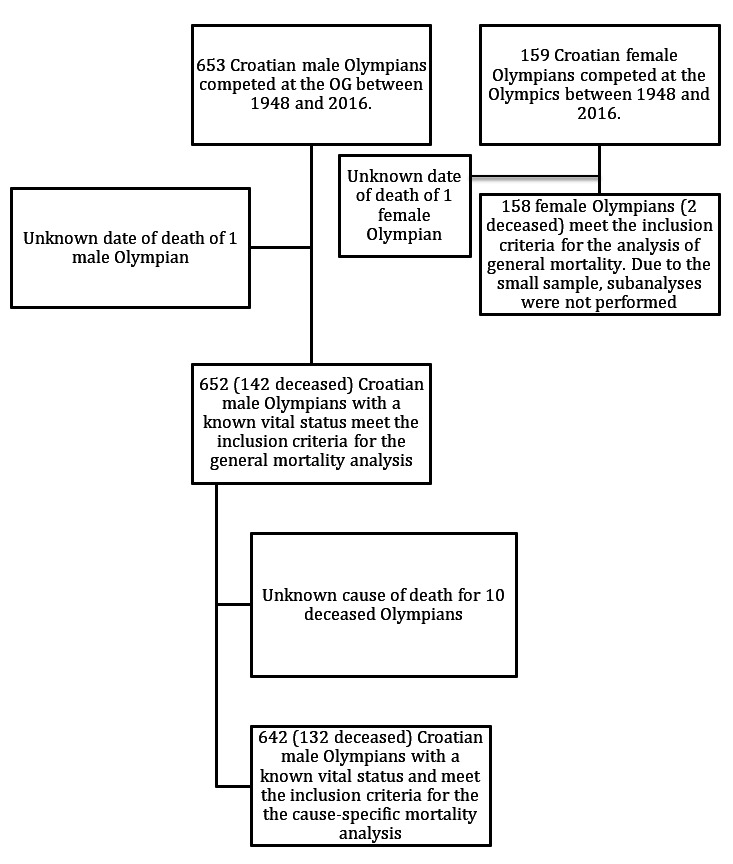
Study flow-chart.

To investigate overall and cause-specific mortalities at different ages, athletes were classified into ten-year age groups: 15-24, 25-34, 35-44, 45-54, 55-64, 65-74, and 75+ years. A broader age group, 15-44 years, was also used, reflecting the typical duration of most athletes' sporting careers. To investigate differences in overall and cause-specific mortality over time, the follow-up period was divided, similarly to previous research (25), into four monitoring periods: 1948-1986, 1987-1996, 1997-2006, and 2007-2016. The study workflow is illustrated in Figure 3.

### Ethics and quality aspects

The research was approved by the Ethics Committee of the Faculty of Medicine, University of Zagreb (380-59-10106-19-111/256) and the Ethics Committee of the Croatian Institute of Public Health (381-14-140-19-1). All data were handled with the utmost care and in accordance with international ethical standards, including the principles of the Nuremberg Code and the latest revision of the Declaration of Helsinki. As stipulated in Recital 27 of the General Data Protection Regulation (GDPR, Regulation (EU) 2016/679), the Regulation does not apply to the personal data of deceased persons; therefore, no GDPR provisions were breached.

### Data management and statistics

The overall and cause-specific mortality rates of Croatian Olympians and those of the general population, standardized by age, sex, and period, were compared using the standardized mortality ratio (SMR). The 95% confidence interval (CI) for the SMR was calculated using Byar's approximation, a method based on the Poisson distribution suitable for low to moderate number of observed events. The number of expected deaths in the general population was calculated by summing the products of the age-, sex-, and period-specific general population mortality rates and the athletes' corresponding person-years at risk for each age group and year throughout the observation period. The SMR is derived by dividing the number of observed deaths in the investigated cohort by the number of expected deaths based on the rates in the general population standardized by age and time period. A *P* value below 0.05 was considered statistically significant. Person-years were calculated as the sum of each athlete’s follow-up time and used to account for different lengths of follow-up among subgroups (Table 1). Statistical analyses was performed using R software (version 4.2.0; R Core Team, Vienna, Austria) and the open-source, web-based OpenEpi statistical tool (version 3.01; Emory University, Atlanta, Georgia, USA) (40,41).

**Table 1:** Data on male and female cohort characteristics

	Female n = 158	Male (general mortality analysis) n = 652	Male (cause-specific mortality analysis) n = 642
Person-years			
total	2751.8	18 595.4	18 128.0
15-24 age	439.1	1063.3	1051.8
25-34 age	1029.3	4995.8	4925.8
35-44 age	593.9	4475.6	4375.6
45-54 age	361.8	3348.9	3248.9
55-64 age	205.0	2405.0	2316.6
65-74 age	84.1	1526.5	1451.6
75+ age	38.6	780.3	758.2
1948-86 period	432.7	6792.2	6432.8
1987-96 period	423.0	3201.0	3134.7
1997-06 period	719.0	4012.2	3974.7
2007-16 period	1177.1	4590.0	4585.8
Age of first Olympic Games appearance, y*	22.0 (20.0-26.0)	24.0 (22.0- 27.0)	24.0 (22.0- 27.0)
Follow-up time, y*	15.0 (5.0-29.0)	26.5 (13.0- 45.0)	25.0 (13.0- 45.0)
Age of death, years	69.0 (60.0-78.0)^†^	72.5 (60.3-80.8)*	71.0 (60.0-81.0)*

*results are shown median (interquartile range).

†since 2 deaths were observed, results are shown as mean (range between ages of recorded deaths).

## RESULTS

### Male population

**Figure 4:**
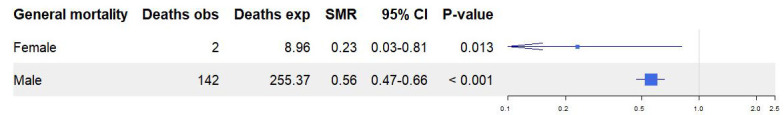
Male and female general mortality analysis (obs – observed, exp – expected; SMR – standardized mortality ratio; CI – confidence interval).

**General male mortality.** During the follow-up, 142 Croatian male Olympians died. The expected number of deaths in the Croatian male general population was 255.37. The SMR was 0.56 (95% CI 0.47-0.66, *P* < 0.001) (Figure 4).

**Figure 5:**
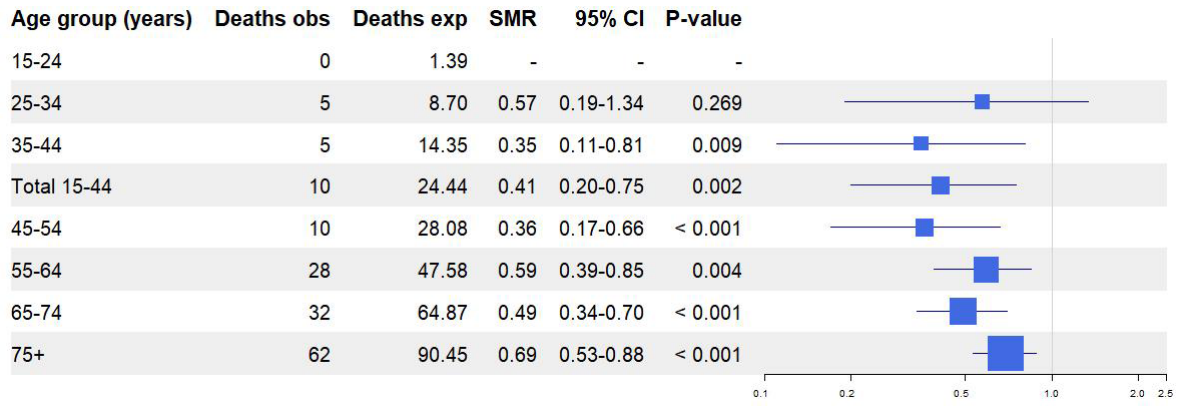
Overall male mortality by age group (obs – observed, exp – expected; SMR – standardized mortality ratio; CI – confidence interval).

**Figure 6:**
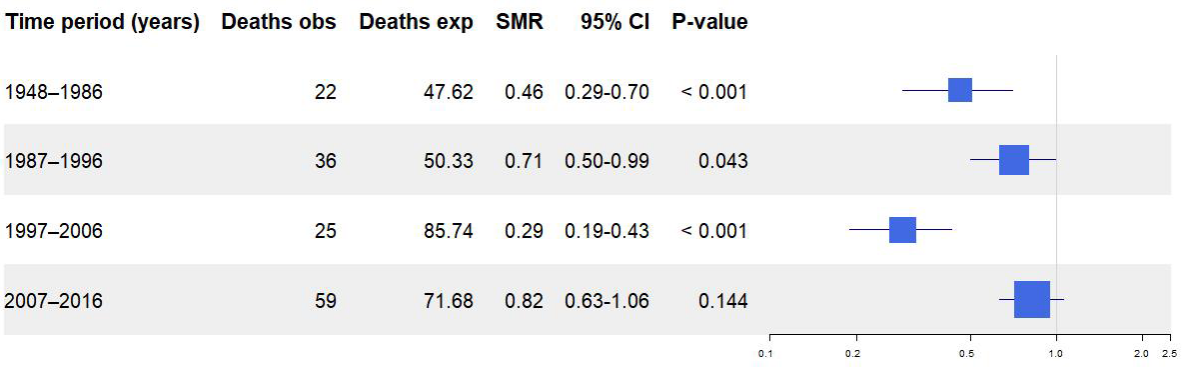
: General male mortality by period (obs – observed, exp – expected; SMR – standardized mortality ratio; CI – confidence interval).

No Olympian died between the ages of 15 and 24. Mortality was significantly reduced in all other age groups, except for the 25-34 group. In the 15-44 group, which covers the career span of most Olympians, the mortality reduction was significant (Figure 5). The SMR reductions for the 1948-1986, 1987-1996, and 1997-2006 periods were significant (Figure 6).

**Figure 7:**
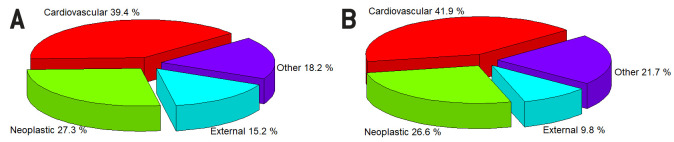
Causes of death among Croatian male Olympians (**A**) and in the Croatian general male population (**B**).

**Male mortality by causes of death.** Of 642 athletes, 132 died. Ten deceased Olympians, for whom the cause of death was unknown, were excluded from the specific mortality analysis. Overall, 52 (39.4%) Olympians died from cardiovascular causes, 36 (27.3%) from neoplastic causes, 20 from external causes (18.2%), and 24 (15.2%) from other causes. Observed and expected causes of death are shown in Figures 7A and 7B. Information on the causes of death for 50% of Olympians was obtained from official registers, and 50% by VA-based interview. Data on athletes meeting the inclusion criteria for cause of death analysis are presented in Table 1.

**Figure 8:**
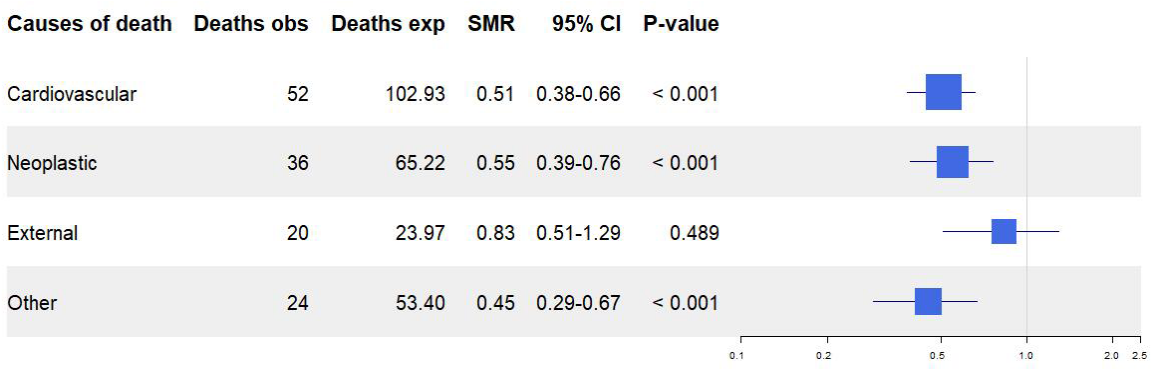
Cause-specific male mortality (obs – observed, exp – expected; SMR – standardized mortality ratio; CI – confidence interval).

Mortality was significantly reduced for cardiovascular (SMR 0.51; 95% CI 0.38-0.66; *P* < 0.001), neoplastic (SMR 0.55; 95% CI 0.39-0.76; *P* < 0.001), and other causes of death (SMR 0.45; 95% CI 0.29-0.67; *P* < 0.001). Mortality from external causes of death did not significantly differ from the general population (SMR 0.83; 95% CI 0.51-1.29; *P* = 0.489). Cause-specific mortality data analysis is provided in Figure 8.

**Figure 9:**
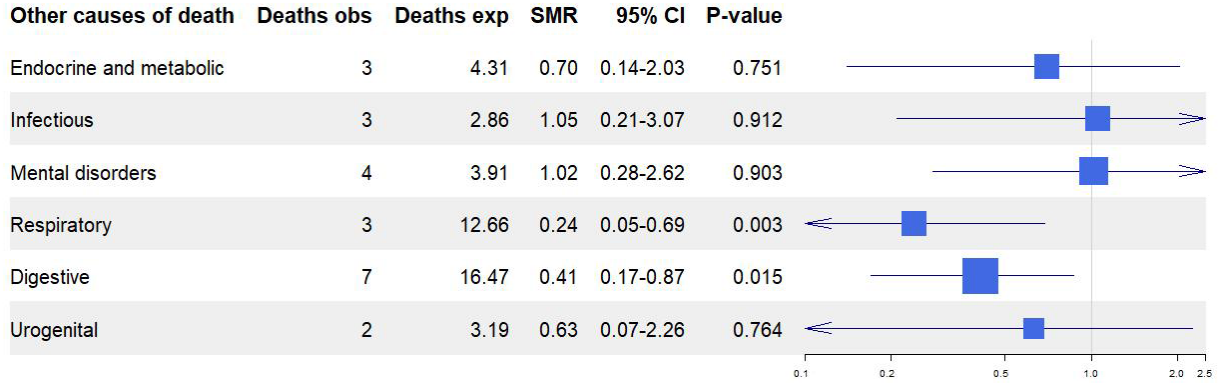
Other causes of death in male mortality subanalysis (obs – observed, exp – expected; SMR – standardized mortality ratio; CI – confidence interval). Two athletes died from unknown causes of death. SMR on unknown causes was not calculated.

Regarding other causes of death (Figure 9), deaths from various disease groups were recorded, including endocrine, metabolic, infectious, mental, digestive, respiratory, and urogenital diseases. Mortalities from respiratory (SMR 0.24; 95% CI 0.05-0.69; *P* = 0.003) and digestive (SMR 0.42; 95% CI 0.17-0.87; *P* = 0.015) causes were significantly reduced (Figure 7). Given the relatively small number of deaths in the age groups 15-24, 25-34, and 35-44, these groups were analyzed as a single group (age group 15-44). Cardiovascular mortality was significantly reduced in the age groups 45-54, 65-74, and 75+ years. Mortality reduction for other age groups was not significant (Table 2).

**Table 2:** Cause-specific mortality by age groups in years for men*

	Age group				
Mortality	15-44	45-54	55-64	65-74	75+
Cardiovascular					
observed/ expected deaths	3/3.44	1/7.58	9/16.46	9/27.44	30/48.01
SMR (95% CI)	0.87 (0.18-2.59)	0.13 (0.001-0.73)	0.55 (0.25-1.04)	0.33 (0.15-0.62)	0.62 (0.42-0.89)
*P* value	0.899	0.009	0.069	<0.001	0.007
Neoplastic					
observed/ expected deaths	0/3.00	3/7.35	7/15.71	14/19.85	12/19.31
SMR (95% CI)	-	0.41 (0.08-1.19)	0.45 (0.18-0.92)	0.71 (0.39-1.18)	0.62 (0.32-1.07)
*P* value	-	0.130	0.024*	0.221	0.106
External					
observed/ expected deaths	7/10.21	6/4.49	5/3.48	1/2.63	1/3.16
SMR (95% CI)	0.69 (0.27-1.41)	1.33 (0.49-2.91)	1.44 (0.46-3.35)	0.38 (0.005-2.12)	0.32 (0.004-1.76)
*P* value	0.403	0.589	0.539	0.521	0.350
Other					
observed/ expected deaths	0/7.17	0/7.68	5/9.96	5/11.37	14/17.22
SMR (95% CI)	-	-	0.50 (0.16-1.17)	0.44 (0.14-1.03)	0.81 (0.33-1.36)
*P* value	-	-	0.137	0.060	0.528

*Abbreviations: SMR – standardized mortality ratio; CI – confidence interval.

Concerning neoplastic causes of death, no fatalities were observed in the youngest age groups (15-44 years). Mortality was significantly reduced specifically in the 55-64 age group. For other age groups, the reduction in neoplastic mortality was not significant. Regarding other causes of death, no fatalities were observed in the age groups 15-44 and 45-54. For the other age groups, the reduction in mortality from other causes of death was not significant.

For the 1948-1986 period, the mortality from other causes of death was significantly reduced. In the 1987-1996 period, cardiovascular mortality was significantly reduced. During the 1997-2006 period, cardiovascular, neoplastic, and other causes of death were significantly reduced. There were no deaths from external causes in the 2007-2016 period (Table 3).

**Table 3:** Cause-specific mortality by time-periods for men*

	Time-period			
Mortality	1948-1986	1987-1996	1997-2006	2007-2016
Cardiovascular				
observed/ expected deaths	9/12.46	11/19.98	4/34.94	28/35.56
SMR (95% CI)	0.72 (0.33-1.37)	0.55 (0.27-0.99)	0.11 (0.03-0.29)	0.79 (0.52-1.14)
*P* value	0.408	0.043	<0.001	0.231
Neoplastic				
observed/ expected deaths	4/9.08	7/12.32	6/19.88	19/23.93
SMR (95% CI)	0.44 (0.12-1.13)	0.57 (0.23-1.17)	0.30 (0.11-0.66)	0.79 (0.48-1.24)
*P* value	0.104	0.152	<0.001	0.367
External				
observed/ expected deaths	6/8.65	10/5.49	4/4.74	0/5.10
SMR (95% CI)	0.69 (0.25-1.51)	1.82 (0.87-3.35)	0.84 (0.23-2.16)	-
*P* value	0.480	0.107	0.975	-
Other				
observed/ expected deaths	1/13.77	6/9.64	6/14.12	11/15.88
SMR (95% CI)	0.07 (0.001-0.40)	0.62 (0.23-1.36)	0.42 (0.16-0.92)	0.69 (0.35-1.24)
*P* value	<0.001	0.308	0.027	0.266

*Abbreviations: SMR – standardized mortality Ratio; CI – confidence interval.

### Female population

Of 159 Croatian female athletes, 158 met the inclusion criteria for the general mortality analysis. During the follow-up, two deaths were observed (Figure 1). The expected number of deaths in the Croatian female general population was 8.96. The SMR was 0.23 (95% CI 0.03-0.81, *P* = 0.013) (Table 1). No age- or cause-specific analyses were performed due to the limited number of deaths.

## DISCUSSION

This article is one of the most extensive pieces of research on Olympic athletes with cause-specific mortality analysis. Furthermore, it represents the most extensive study of Croatian elite athletes' mortality. It showed a significant reduction in overall mortality, as well as mortality from cardiovascular, neoplastic, and other causes of death for Croatian male Olympians compared with the general population. Reduction in mortality from other causes of death was driven by reduction in mortality from digestive and pulmonary causes. There was no significant difference in mortality from external causes of death. Based on a larger sample, the results largely confirm the findings from a previous study on Croatian Olympic medalists (24). In the previous study, athletes had significantly lower overall and cardiovascular mortality, while reductions in mortality from neoplastic and other causes of death were not significant, possibly due to a smaller sample size (2).

In sum, our results speak against the hypothesis that competitive sports seriously harm health. Previous studies also found reduced general mortality in French, Japanese, US, and Polish Olympians, international Olympic medal winners, and a large meta-analysis of smaller studies of elite athletes (17,21,26,27,29). Studies on French and US Olympians found reduced mortalities from cardiovascular, neoplastic, digestive, respiratory, and endocrine causes of death (21,26). On the other hand, studies of German and Danish Olympic athletes did not find a significant overall mortality reduction compared with the general population (30,31). The study of Danish athletes failed to find a significant cause-specific mortality reduction for cardiovascular, neoplastic, or other causes of death (31). These findings can partly be explained by Danish and German residents’ above-average healthy lifestyles. Only 19% of Croatian adult men meet the WHO-recommended physical activity level of at least 75 minutes of moderate physical activity per week, compared with 72% and 46% of men in Denmark and Germany, respectively (42).

Despite the beneficial effects of physical activity on the cardiovascular system, the connection between intense physical activity and certain cardiovascular diseases is the subject of numerous studies. Some studies find intense physical activity associated with atrial and ventricular arrhythmias, myocardial fibrosis, right ventricular systolic dysfunction, and atherosclerosis with coronary artery calcification, whereas others do not (13,43-50). Our results imply that the potentially harmful cardiovascular mechanisms of vigorous training are not significant enough to deny the general health benefits of physical activity. In addition, the anti-inflammatory effect of physical activity has an important role in the risk-reduction of malignancies, as well as metabolic and other chronic diseases. Furthermore, most athletes adhere to healthier lifestyles than the general population (10,51,52).

Regarding age group mortality, all age groups' general moralities were significantly reduced, except in the 25-34 group (possibly due to a relatively small number of events observed among Olympians and expected in the general population of that age group). In the 15-44 age group, which covers the sports careers of most Olympians, athletes had significantly lower general, neoplastic, and other cause mortality with no significant difference in cardiovascular mortality. A study on French Olympians with a larger sample reported a significant overall mortality reduction, but did not report cause-specific mortality (21).

Cardiovascular mortality was significantly lower than in the general population in the 45-54, 65-74, and 75+ age groups, but not in the 55-64 age group. This finding might be explained by some Croatian Olympians having abandoned healthy habits after the end of their sports careers, which was hypothesized in the study on Croatian Olympic medalists as well (24). Our study did not seek data to confirm this assumption. Conversely, neoplastic mortality reduction was significant only in the 55-64 age group, while there was no significant reduction for other groups.

Regarding time periods, mortality was significantly reduced for the 1948-1986, 1987-1996, and 1997-2006 periods. The 1987-1996 period includes the Croatian Homeland War (1991-1995). Although overall SMR was significantly reduced, it was higher than in previous and subsequent periods. A marginally significant reduction in mortality was recorded only in the cardiovascular group of causes of death. In addition, SMR for external causes of death was greater than 1, though it was not significant. One Croatian male Olympic athlete died in a military operation during the Croatian Homeland War. Although both Croatian Olympians and the general Croatian population were affected by the war, it is impossible to rule out direct or indirect effects of the war on mortality outcomes. The lack of significance in mortality reduction for the 2007-2016 period should be interpreted with caution. Given the heterogeneity of this broad age group, the observed effect could also reflect statistical fluctuation rather than a consistent underlying pattern.

Croatian female Olympic athletes had significantly lower overall mortality than the general female population. Mortality analyses by age, period, and cause of death were not performed due to a small number of deaths. Similar problems exist in other epidemiological studies on competitive female athletes (10,29). Overall, female athletes are much less frequently represented in epidemiological studies than male athletes. Nonetheless, a meta-analysis found a reduction in female athletes’ mortality compared with the general population (10). Mortality was significantly reduced compared with the general population in French and US female Olympians, while in Polish Olympians the reduction was not significant (21,26,29). A study showed that US female Olympians were at a lower risk of death than the general population from cardiovascular, neoplastic, external, endocrine, respiratory, and digestive causes (26). Nonetheless, high-quality studies on female athletes are lacking, and further research is needed, especially for cause-specific mortality.

This research has several limitations. First, the reliability of data on causes of death may vary between data from official registers and data obtained by interview according to VA principles. However, a more reliable method of data collection was not available. Furthermore, the sample size for some analyses was not large enough to produce meaningful conclusions (eg, for cause-specific analysis of female deaths). In categories with fewer than 10 deaths, results should be interpreted with caution because small numbers introduce statistical instability. Despite this limitation, we believe the findings are worth reporting as they provide rare and valuable insights into the long-term health outcomes of this unique population. Selection bias is another limitation. The general population consists of a certain number of people with chronic or genetic conditions who have a smaller probability of becoming elite athletes than the mostly healthy individuals in the competitive athletes’ cohort. Another shortcoming is the unavailability of additional data on the athlete population that could influence the results. This primarily refers to tobacco smoking, consumption of alcohol and narcotic drugs, doping abuse, nutrition, comorbidities, medical check-ups, socioeconomic status, body proportions, and physical activity level after retirement from sports (16,51-53).

In conclusion, Croatian male Olympians have reduced overall, cardiovascular, neoplastic, digestive, and respiratory mortality rates compared with the general population. Overall, Olympians’ mortality advantage was preserved during most of the follow-up period and across most age groups. Croatian female Olympians have lower overall mortality compared with the general Croatian female population. Further research on a larger sample and with more covariates measured is needed, especially for female competitive athletes.
